# Testing of Exchange-Correlation Functionals of DFT for a Reliable Description of the Electron Density Distribution in Organic Molecules

**DOI:** 10.3390/ijms232314719

**Published:** 2022-11-25

**Authors:** Małgorzata Domagała, Mirosław Jabłoński, Alina T. Dubis, Manfred Zabel, Arno Pfitzner, Marcin Palusiak

**Affiliations:** 1 Faculty of Chemistry, University of Lodz, Pomorska 163/165, 90-236 Lodz, Poland; 2 Faculty of Chemistry, Nicolaus Copernicus University in Toruń, Gagarina 7, 87-100 Toruń, Poland; 3 Faculty of Chemistry, University of Białystok, Ciołkowskiego 1K, 15-245 Białystok, Poland; 4 Institute of Inorganic Chemistry, University of Regensburg, Universitätsstr. 31, 93040 Regensburg, Germany

**Keywords:** DFT, exchange-correlation functional, Jacob’s Ladder, electron density, QTAIM, benchmark

## Abstract

Researchers carrying out calculations using the DFT method face the problem of the correct choice of the exchange-correlation functional to describe the quantities they are interested in. This article deals with benchmark calculations aimed at testing various exchange-correlation functionals in terms of a reliable description of the electron density distribution in molecules. For this purpose, 30 functionals representing all rungs of Jacob’s Ladder are selected and then the values of some QTAIM-based parameters are compared with their reference equivalents obtained at the CCSD/aug-cc-pVTZ level of theory. The presented results show that the DFT method undoubtedly has the greatest problems with a reliable description of the electron density distribution in multiple strongly polar bonds, such as C=O, and bonds associated with large electron charge delocalization. The performance of the tested functionals turned out to be unsystematic. Nevertheless, in terms of a reliable general description of QTAIM-based parameters, the M11, SVWN, BHHLYP, M06-HF, and, to a slightly lesser extent, also BLYP, B3LYP, and X3LYP functionals turned out to be the worst. It is alarming to find the most popular B3LYP functional in this group. On the other hand, in the case of the electron density at the bond critical point, being the most important QTAIM-based parameter, the M06-HF functional is especially discouraged due to the very poor description of the C=O bond. On the contrary, the VSXC, M06-L, SOGGA11-X, M06-2X, MN12-SX, and, to a slightly lesser extent, also TPSS, TPSSh, and B1B95 perform well in this respect. Particularly noteworthy is the overwhelming performance of double hybrids in terms of reliable values of bond delocalization indices. The results show that there is no clear improvement in the reliability of describing the electron density distribution with climbing Jacob’s Ladder, as top-ranked double hybrids are also, in some cases, able to produce poor values compared to CCSD.

## 1. Introduction

Due to the relatively low computational cost and generally good accuracy of the results obtained, density functional theory (DFT) [1,2,3] is currently the most frequently used method of describing electronic structure of molecules in computational chemistry [4]. The known problem of this method is the lack of the exact form of the so-called exchange-correlation functional, as a result of which it is necessary to use its worse or better approximations [5,6]. Unfortunately, one could say that at present there are as many exchange-correlation functionals as ants in an anthill, which leads to the situation that we currently have hundreds of them, and choosing the most appropriate one for a given problem is one of the biggest concerns when using DFT. Therefore, benchmark calculations in which the reliability of DFT functionals is tested are extremely important [5,6,7,8,9,10,11,12,13,14,15,16,17,18,19,20,21,22,23,24,25,26,27,28,29,30,31,32,33,34,35,36,37,38,39,40,41,42,43,44,45,46,47,48,49,50,51]. However, the bottleneck here is the enormous amount of calculations to be made. It is enough to mention that when choosing *A* functionals, *B* properties (parameters), *C* molecules, and possibly *D* basis sets, the size of such calculations is A×B×C×D. It is therefore clear that such benchmarking calculations must be suppressed by greatly reducing all or some of the numbers *A*, *B*, *C*, and *D*. In general, this is achieved by limiting ourselves to few parameters of interest only and using only one, but a fairly large and therefore reliable, basis set. Additionally, calculations can be made for one representative, and in some sense any, molecule. The complexity of the problem can be easily outlined with reference to the benchmark review article by Sousa et al. [21] from 2007, in which they stated that the ranking of DFT functionals depends on many factors, e.g., the set of tested functionals, the set of systems, the set of investigated properties and the basis set(s) used, and concluded that there is no universal exchange-correlation functional. Although this is still undoubtedly true even after 15 years, more and more recent studies show that the overall reliability of exchange-correlation functionals is consistent with the so-called Jacob’s Ladder, i.e., the adopted hierarchy of functionals according to their generation [52,53]. Thus, gradient functionals are generally better than local functionals, meta-gradient functionals and hybrid functionals are preferable to them, and double hybrid functionals are the best [6] (see the Crystal Structure and Methodology section for an explanation of the respective types of exchange-correlation functionals). Although this general principle was confirmed in the latest benchmark studies by Brémond et al. [50], it should be emphasized that a good performance for energy-based quantities does not go hand in hand with a good description of electron-density-based quantities [36,37,39,50]. Indeed, highly parametrized exchange-correlation functionals have been proven to give reliable values for energy-based parameters, but not necessarily for electron density-based quantities. Moreover, functionals with no or poor parametrization give a fairly good density, the quality of which actually increases gradually as one climbs up Jacob’s Ladder [36,37,39,50].

It is therefore clear that benchmarking must be significantly limited. Even the most extensive review article by Mardirossian and Head-Gordon, in which as many as 200 exchange-correlation functionals and a whole multitude of physico-chemical quantities were tested [6], did not refer to the reliability of the exchange-correlation functionals in describing the electron density distribution (EDD), which in turn can be well represented by some QTAIM-based parameters. However, QTAIM (i.e., quantum theory of atoms in molecules [54]) is still one of the most widely used theoretical methods for describing a wide variety of bonds and interactions.

This article is aimed at testing selected exchange-correlation functionals in terms of describing EDD being represented by some QTAIM-based parameters. It is worth recalling that similar benchmark calculations, but for basis sets, were once performed by two of us (M.J. and M.P.) [55,56]. When it comes to testing various exchange-correlation functionals for the quality of the EDD description represented by some QTAIM-based parameters, this type of benchmark computation was first performed by Tognetti and Joubert in 2011 [26]. At that time, they used 10 functionals representing all rungs of Jacob’s Ladder except the highest one, which is the most computationally costly double hybrids, and their performance was checked for five local QTAIM-based parameters. Only recently, Brémond et al. performed similar benchmark calculations for as many as 29 functionals and hundreds of bond critical points in molecules belonging to various databases [50].

**Figure 1:**
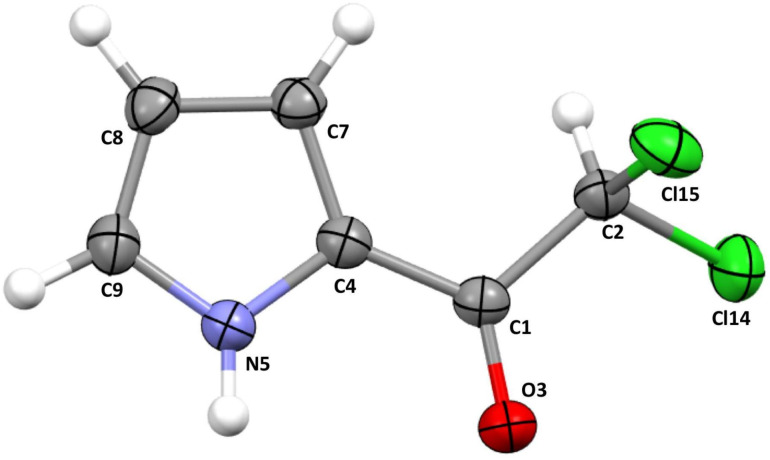
Crystallographic structure of 2,2-dichloro-1-(1H-pyrrol-2-yl)ethan-1-one (CCDC refcode: 2120678). Atomic displacement ellipsoids are drawn with a 50% probability level. The labels of individual atoms used in the article are also shown.

It should be emphasized that conducting this type of research, even for only one reference molecule, requires establishing a certain strategy for selecting the structure for this molecule. One possibility is to optimize the geometry of this molecule separately for each of the tested functionals, and then perform QTAIM calculations for these certainly different geometries. Consequently, these different geometries result in different EDDs. Moreover, within this approach, differences in descriptions of the geometries are mixed with differences in descriptions of EDDs. For this reason, in this work we adopted the second possible strategy, namely, the performance of benchmark calculations for the same arbitrarily chosen reference structure and a high-quality basis set. An important issue that arises here is the correct choice of this reference structure. A good solution would perhaps be the use of the highest possible method to obtain the reference geometry. However, a limitation connected with computational possibilities will always force the compromise between the quality of calculations and the size of the system. Our goal is to test EDD in a real molecular system consisting of various atoms, connected via different types of bonds. Therefore, the mentioned limitation does not allow to obtain a computationally satisfactory reference geometry. Therefore, we naturally directed our attention towards experimental data. For this purpose, we use the good quality X-ray crystal structure of 2,2-dichloro-1-(1H-pyrrol-2-yl)ethan-1-one [57] (see the “Crystal Structure and Methodology” section for details) that we solved (see Figure 1).

The 2,2-dichloro-1-(1H-pyrrol-2-yl)ethan-1-one molecule consists of various types of atoms connected by different bonds. These atoms are relatively small and thus give the opportunity to utilize computationally more advanced post-SCF calculations in order to obtain a high-quality reference EDD. Additionally, this molecule is planar due to bonding character and partial intramolecular π-electron delocalization. Therefore, it can be expected that its solid phase and gas phase geometries will not be significantly different due to possible packing effects. The use of geometry from the X-ray experiment has yet another justification. Currently the state-of-the-art X-ray analysis allows to experimentally estimate details of EDD in interatomic space; however, the standard procedure is that experimental results are confronted with theoretical EDD produced for geometry from the crystal (see, for instance, ref. [58] and citations therein). In that context, our analysis gains potentially additional important application, namely, an indication of the DFT functional which reproduces EDD most reliably.

## 2. Results and Discussion

As already mentioned in the Methodology, the electron density distribution (EDD) is probed by the most commonly used QTAIM parameters computed either at the bond critical point (BCP) or ring critical point (RCP): the electron density (ρ), Laplacian of the electron density ($\nabla^{2}\rho$), and total electronic energy density (*H*). Additionally, in the case of bonds, the delocalization index (δ) was also determined. The analysis of the impact of the theoretical method on EDD in BCP will be presented in the first subsection, and in RCP in the second. The impact of the method on the value of the bond delocalization index will be presented in the third subsection.

### 2.1. Testing of DFT Functionals for the Electron Density Distribution in Bond Critical Points

**Figure 2:**
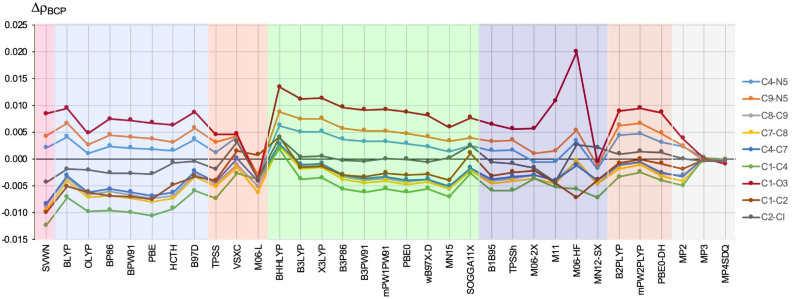
The difference (in relation to the CCSD method) in the value (in a.u.) of the electron density determined at the bond critical point of the bond shown on the right side of the figure. The different types of methods are represented by a colored background: LDA—pink, GGA—blue, M-GGA—orangeish, H-GGA—green, HM-GGA—violet, DH-GGA—beige, and perturbational methods—gray.

Figure 2 shows the difference between the electron density value determined for the BCP ($\rho_{\mathrm{BCP}}$) of a given bond within the given method and the reference CCSD method, i.e., $\Delta \rho_{\mathrm{BCP}}$ = $\rho_{\mathrm{BCP}}$(method) − $\rho_{\mathrm{BCP}}$(CCSD). Thus, a positive value for $\Delta \rho_{\mathrm{BCP}}$ indicates an overestimation, and a negative value for $\Delta \rho_{\mathrm{BCP}}$ indicates an underestimation relative to the reference value for CCSD.

First, it should be noted that the obtained $\Delta \rho_{\mathrm{BCP}}$ values vary considerably depending on the bond under consideration. Importantly, the greatest discrepancies concern the double and highly polarized C1=O3 bond (see also Figure 1), for which the obtained $\Delta \rho_{\mathrm{BCP}}$ values are generally positive, indicating an overestimation of the $\rho_{\mathrm{BCP}}$ value in relation to CCSD. By far, the largest error in relation to the CCSD value was obtained for the M06-HF functional (0.020 a.u.). Then, functionals BHHLYP (0.013 a.u.), B3LYP, X3LYP, and M11 (0.011 a.u.) followed. Quite the opposite, of all the considered functionals, MN12-SX performed the best, giving a negligible error value of only −0.001 a.u. A clearly greater error in the value of $\rho_{\mathrm{BCP}}^{C=O}$, but also a negative one (−0.004 a.u.), was obtained by M06-L. It is worth mentioning that both of these functionals, i.e., MN12-SX and M06-L, were found to be among the best for general purposes [5]. Nevertheless, for all other functionals, the obtained $\Delta \rho_{\mathrm{BCP}}^{C=O}$ values were greater than 0.005 a.u. showing that DFT has a particular problem with accurately describing EDD in this bond. The high sensitivity of the EDD of the double and strongly polarized C=O bond to the applied methodology was already noticed earlier [56]. Interestingly, the GGA (0.005–0.009 a.u.) and M-GGA (ca. ±0.005 a.u.) functionals generally performed better than the H-GGA (0.006–0.013 a.u) functionals (HM-GGA gave both the greatest and the lowest $\Delta \rho_{\mathrm{BCP}}^{C=O}$ values) and double hybrids (0.009 ca.).

Significantly smaller, but also generally positive, deviations were obtained for both C–N bonds (i.e., C4–N5 and C9–N5) from the ring. This time, M06-2X was the best (−0.0007 and 0.0009 a.u. for C4–N5 and C9–N5, respectively). Interestingly, the M11 functional, which gave one of the largest errors in $\rho_{\mathrm{BCP}}^{C=O}$, was only slightly worse (−0.0005 and 0.0015 a.u., respectively). For the C4–N5 bond, negligible error values were also obtained by OLYP (0.0010 a.u.) and TPSS (0.0011 a.u.) and only slightly greater for MN15 (0.0014 a.u.), B1B95 (0.0016 a.u.), and TPSSh (0.0016 a.u.), while for the C9–N5 bond, the MN12-SX functional performed very well (−0.0013 a.u.). On the other hand, again, the BHHLYP functional performed the worst (0.0062 and 0.0087 a.u. for C4–N5 and C9–N5, respectively). Only slightly worse were B3LYP and X3LYP (0.0050 and 0.0075 a.u. for C4–N5 and C9–N5, respectively). Interestingly, the B2-PLYP and mPW2-PLYP double hybrids also performed poorly (ca. 0.0045 a.u. for C4–N5 and ca. 0.0065 for C9–N5), while the third double hybrid, PBE0-DH, produced significantly smaller errors (0.0031 and 0.0048 a.u., respectively).

At the other extreme, i.e., with $\Delta \rho_{\mathrm{BCP}}\;<\;0$, the greatest error was obtained for the nonpolar C1–C4 bond, which as a result of electron density delocalization is intermediate between single and double (Figure 1). In this case, the largest deviation was obtained for the local SVWN functional (−0.013 a.u.) and all GGA functionals (ca. −0.010 a.u.) except BLYP and B97-D, which gave slightly smaller errors (−0.007 and −0.006 a.u., respectively). For the other functionals, the description of EDD in this bond is clearly better. In particular, VSXC (−0.0028 a.u.), SOGGA11-X (−0.0027 a.u.), and mPW2-PLYP (−0.0025 a.u.) performed the best. Interestingly, BHHLYP as the only functional gave a positive error, which was even slightly smaller (0.0018 a.u.). Our results show well that density functionals may have a particular problem with the correct description of EDD not only in highly polarized double bonds but also in bonds that are formally nonpolar but associated with large electron delocalization. This result should be seen as a warning when describing organic compounds. However, the situation is somewhat similar in the case of the MP2 method, because, although in general, clearly smaller than for DFT, the highest values of $\Delta \rho_{\mathrm{BCP}}$ were also obtained for the bonds C1=O3 and C1–C4 (0.0038 and −0.0050 a.u., respectively). On the contrary, the difference to CCSD for MP3 and MP4SDQ is negligible.

The $\Delta \rho_{\mathrm{BCP}}$ errors for the remaining bonds, especially for both C–Cl, are much lower, although SVWN, M11 (both −0.004 a.u.), and BHHLYP (0.004 a.u.) gave clearly greater errors. Importantly, all the H-GGA functionals (but BHHLYP and SOGGA11-X) performed very well, with $\Delta \rho_{\mathrm{BCP}}^{C-\mathrm{Cl}}$ value close to zero. The HCTH, B97-D, B1B95, TPSSh, and B2-PLYP functionals were only slightly worse (ca. −0.001 a.u.).

Tognetti and Joubert reported [26] that the GGA and M-GGA functionals give too small $\rho_{\mathrm{BCP}}$ values and that adding the exact exchange, that is the use of the H-GGA and HM-GGA functionals, remedies this defect somewhat. Our results confirmed this, but differences between the values obtained using the GGA or M-GGA functionals and their hybrid counterparts are rather small. For example, for the C1–C4 bond, we obtained the following values (in a.u.) of $\rho_{\mathrm{BCP}}^{C1-C4}$: BLYP (0.295) < B3LYP (0.298), BP86 (0.292) < B3P86 (0.296), BPW91 (0.292) < B3PW91 (0.296), PBE (0.291) < PBE0 (0.296), TPSS (0.295) < TPSSh (0.296). For C–Cl the respective values are: BLYP (0.193) < B3LYP (0.195), BP86 (0.192) < B3P86 (0.194), BPW91 (0.192) < B3PW91 (0.194), PBE (0.192) < PBE0 (0.195), TPSS (0.193) < TPSSh (0.194), and for C=O: BLYP (0.409) < B3LYP (0.410), BP86 (0.407) < B3P86 (0.409), BPW91 (0.406) < B3PW91 (0.408), PBE (0.406) < PBE0 (0.408), TPSS (0.404) < TPSSh (0.405).

Referring to a very recent study by Brémond et al. [50] it is worth paying special attention to the performance of M06-2X and M06-HF, because these HM-GGA functionals gave the smallest and the largest errors for the electron density at critical points, respectively. As can be seen in Figure 2, also, our results show that M06-2X, regardless of the bond, performs fairly well compared to the other functionals. Nevertheless, as our results show, other functionals, and in particular VSXC, M06-L, SOGGA11-X, and MN12-SX, appear to give better or comparable $\rho_{\mathrm{BCP}}$ values, though perhaps not for all bond types. Both M06-L and MN12-SX were among the best general purpose functionals in Peverati and Truhlar’s earlier studies [5], while the SOGGA11-X functional gave the best Fukui functions [38]. Additionally, as shown in Figure 2, the M-GGA functional VSXC also deserves attention. Moreover, TPSS, TPSSh, and B1B95 also give quite good $\rho_{\mathrm{BCP}}$ values, although again perhaps not necessarily for all bond types (Figure 2). All of these functionals were also previously mentioned as some of the best in the EDD description [26,38,39]. Therefore, our results are in line with the previous results and complement them nicely. On the other hand, when it comes to the poor description of $\rho_{\mathrm{BCP}}$ by M06-HF [50], the extremely large error obtained for the strongly polarized C=O bond actually disqualifies this functional, although for some types of bonds (e.g., C7–C8 and C4–C7), it performed very well (Figure 2).

**Figure 3:**
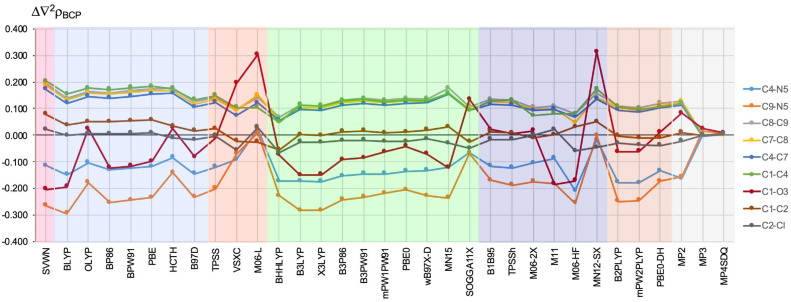
The difference (in relation to the CCSD method) in the value (in a.u.) of the Laplacian of the electron density determined at the bond critical point of the bond shown on the right side of the figure. The different types of methods are represented by a colored background: LDA—pink, GGA—blue, M-GGA—orangeish, H-GGA—green, HM-GGA—violet, DH-GGA—beige, perturbational methods—gray.

The differences in $\nabla^{2}\rho_{\mathrm{BCP}}$ values, i.e., Laplacian of the electron density, are shown in Figure 3.

As the second derivative of the electron density, $\nabla^{2}\rho_{\mathrm{BCP}}$ is a quantity quite sensitive to the adopted methodology, e.g., the basis set used [56]. The current research also confirmed this. Namely, for many bonds, the differences in the obtained values of $\Delta \nabla^{2}\rho_{\mathrm{BCP}}$ are very large. Again, the C=O bond is clearly distinguished for which the values of $\Delta \nabla^{2}\rho_{\mathrm{BCP}}$ can be either significantly negative (e.g., ca. −0.200 a.u. for SVWN, BLYP, and M11) or significantly positive (0.313 a.u. for MN12-SX, 0.302 a.u. for M06-L, 0.195 a.u. for VSXC), so the range of values for this bond is as much as 0.515 a.u. It is noteworthy that, this time, among the worst functionals are M06-L and MN12-SX, which, as discussed earlier, gave quite good $\rho_{\mathrm{BCP}}$ values (see Figure 2).

Large errors, but only negative (with the exception of M06-L only giving a negligible error of 0.010 a.u), characterize the C9–N5 bond and, to a much lesser extent, also C4–N5. The most negative value of $\Delta \nabla^{2}\rho_{\mathrm{BCP}}^{C9-N5}$ was obtained for BLYP (−0.294 a.u.), B3LYP (−0.282 a.u.), and X3LYP (−0.282 a.u.). The next poor performers were SVWN (−0.265 a.u.), M06-HF (−0.255 a.u.), BP86 (−0.253 a.u.), B2-PLYP −0.248 a.u.), mPW2-PLYP (−0.245 a.u.), BPW91 (−0.244 a.u.), and B3P86 (−0.241 a.u.). As one can see, among the rather poor performers for the $\Delta \nabla^{2}\rho_{\mathrm{BCP}}^{C9-N5}$ parameter, there are also double hybrids B2-PLYP and mPW2-PLYP. However, the third DH-GGA, i.e., PBE0-DH, performed significantly better (−0.172 a.u.) and was comparable to the MP2 method (−0.156 a.u.). On the other hand, the smallest values of $\Delta \nabla^{2}\rho_{\mathrm{BCP}}^{C9-N5}$ were obtained by functional MN12-SX, which gave a negligible error of only −0.001 a.u., and the aforementioned M06-L, which was the only one that gave a positive deviation of 0.010 a.u.

From Figure 3, it is clear that all the tested functionals, except VSXC (ca. 0.090 a.u.), BHHLYP (ca. 0.070 a.u.), and M06-HF (ca. 0.070 a.u.), consistently gave a positive $\Delta \nabla^{2}\rho_{\mathrm{BCP}}$ error in the range of ca. 0.100–0.200 a.u. for the C4–C7, C7–C8, C8–C9, and C1–C4 bonds. The first three form the imidazole ring, while the last one is a linker to the –(CO)–CHCl${}_{2}$ group. Thus, they all participate in a conjugated system with a high degree of electron charge delocalization. Together with the previously discussed large errors for the C=O and C–N bonds, this result again demonstrates the difficulty of describing EDD in bonds with large charge delocalization.

**Figure 4:**
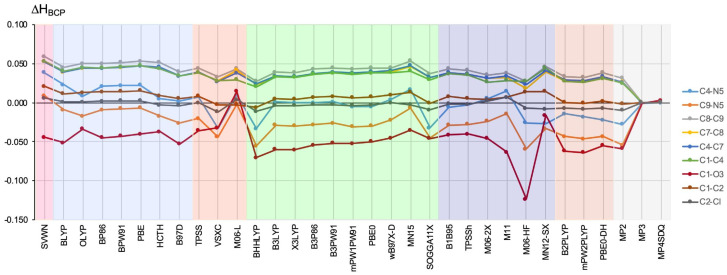
The difference (in relation to the CCSD method) in the value (in a.u.) of the total electronic energy density determined at the bond critical point of the bond shown on the right side of the figure. The different types of methods are represented by a colored background: LDA—pink, GGA—blue, M-GGA—orangeish, H-GGA—green, HM-GGA—violet, DH-GGA—beige, perturbational methods—gray.

In contrast, the $\Delta \nabla^{2}\rho_{\mathrm{BCP}}$ values are significantly lower for C1–C2 and both C–Cl bonds, and therefore for the single bonds. While the local functional SVWN and all GGA functionals gave a significantly smaller error in the $\Delta \nabla^{2}\rho_{\mathrm{BCP}}$ value for C–Cl (ca. 0.000 a.u.), the trend is generally reversed in the case of hybrid and double hybrid functionals (and, in addition, $\Delta \nabla^{2}\rho_{\mathrm{BCP}}^{C-\mathrm{Cl}}$ is generally negative), but the differences for both bond types are small. Nevertheless, it can be seen that BHHLYP, M06-HF, and MN12-SX gave the greatest errors. Interestingly, along with B1B95, the BHHLYP functional gave the best $\Delta \nabla^{2}\rho_{\mathrm{BCP}}$ values in the earlier calculations by Tognetti and Joubert (however, only 10 functionals were tested then) [26]. Our results presented in Figure 4 clearly show, however, that while this functional indeed produced reasonable $\Delta \nabla^{2}\rho_{\mathrm{BCP}}$ values for many types of chemical bonds (e.g., C1–C4, C7–C8, C4–C7), the overall performance of BHHLYP is rather unimpressive.

The obtained $\Delta H_{\mathrm{BCP}}$ values are shown in Figure 4.

Again, it can be seen that the obtained $\Delta H_{\mathrm{BCP}}$ values can be both positive and negative, not only depending on the bond but also for the selected bond (see the bright blue line for the C4–N5 bond or the red line for C1–O3 (i.e., C=O)). In the former case, the $\Delta H_{\mathrm{BCP}}^{C4-N5}$ values are in a wide range, from −0.033 a.u. for VSXC, BHHLYP, and SOGGA11-X, up to 0.038 a.u. for SVWN. However, by far, the largest error value was obtained by the M06-HF functional for the C=O bond (−0.124 a.u.). Much smaller, but also significant, $\Delta H_{\mathrm{BCP}}^{C=O}$ errors were obtained by functionals BHHLYP (−0.071 a.u.), mPW2-PLYP (−0.064 a.u.), M11 (−0.063 a.u.), B2-PLYP (−0.062 a.u.), B3LYP (−0.060 a.u.), and X3LYP (−0.060 a.u.).

On the contrary, the largest positive error (up to 0.060 a.u.) was obtained within the SVWN functional for the C8–C9, C7–C8, C4–C7, and C1–C4 bonds. For these bonds, the VSXC (ca. 0.028 a.u.), BHHLYP (ca. 0.020 a.u.), and M06-HF (ca. 0.025 a.u.) functionals performed the best. The smallest error in the $H_{\mathrm{BCP}}$ value was obtained for both C–Cl bonds, but in this respect, the VSXC and BHHLYP functionals performed the worst (both −0.011 a.u.).

### 2.2. Testing of DFT Functionals for the Electron Density Distribution in a Ring Critical Point

**Figure 5:**
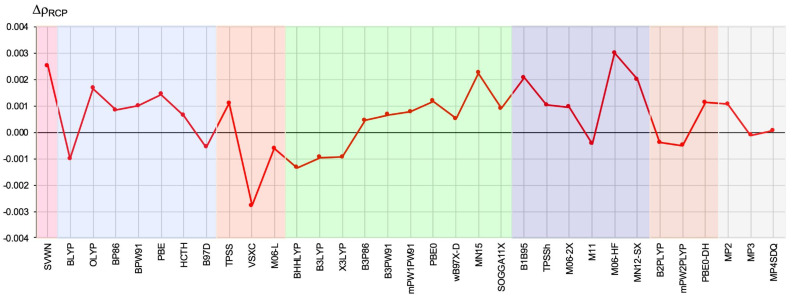
The difference (in relation to the CCSD method) in the value (in a.u.) of the electron density determined at the ring critical point of the 2,2-dichloro-1-(1H-pyrrol-2-yl)ethan-1-one molecule. The different types of methods are represented by a colored background: LDA—pink, GGA—blue, M-GGA—orangeish, H-GGA—green, HM-GGA—violet, DH-GGA—beige, perturbational methods—gray.

Another source of information on the reliability of the description of EDD by means of exchange-correlation functionals of the DFT method may be the values of ρ, $\nabla^{2}\rho$, and *H* obtained in the RCP, which is possible due to the presence of the imidazole ring in the molecule under consideration. The resulting $\Delta \rho_{\mathrm{RCP}}$ values are shown in Figure 5.

Dispersion of the values is considerable and, moreover, depending on the method used, these values may be negative or positive. The largest negative error was obtained for VSXC (−0.0028 a.u.), while the greatest positive errors were obtained for M06-HF (0.0030 a.u.) and SVWN (0.0025 a.u.). On the contrary, the best compliance (≤±0.0005 a.u.) with the CCSD value was obtained for B3P86, ωB97X-D, M11, B2-PLYP, and mPW2-PLYP. However, it is not optimistic that even quite-high-ranked hybrid functionals can give both positive and negative values of $\Delta \rho_{\mathrm{RCP}}$.

**Figure 6:**
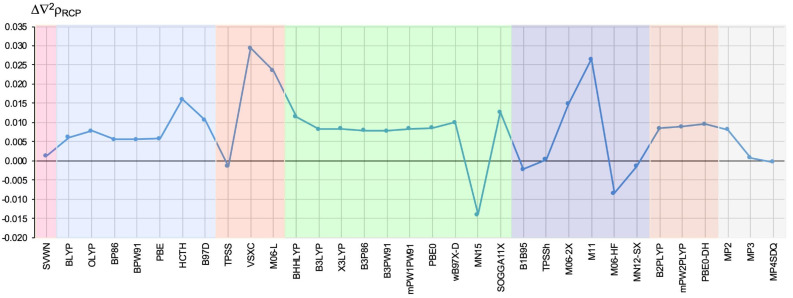
The difference (in relation to the CCSD method) in the value (in a.u.) of the Laplacian of the electron density determined at the ring critical point of the 2,2-dichloro-1-(1H-pyrrol-2-yl)ethan-1-one molecule. The different types of methods are represented by a colored background: LDA—pink, GGA—blue, M-GGA—orangeish, H-GGA—green, HM-GGA—violet, DH-GGA—beige, perturbational methods—gray.

The result of a similar analysis but relating to $\Delta \nabla^{2}\rho_{\mathrm{RCP}}$ is shown in Figure 6.

Again, the scatter of the values obtained is very large, from −0.014 a.u. (for MN15) up to 0.029 a.u. The VSXC and M11 functionals gave the biggest errors (0.029 and 0.026 a.u., respectively) in relation to the CCSD value. On the contrary, TPSSh gave a negligible error of only 0.0004 a.u. Very small errors were also obtained by functionals SVWN (0.0013 a.u.), TPSS (−0.0014 a.u.), MN12-SX (−0.0014 a.u.), and B1B95 (−0.0022 a.u.). A similar error was obtained by the MP3 method (0.0008 a.u.), whereas MP2 performed much worse (0.0082 a.u.).

It is worth noting that in the case of $\Delta \nabla^{2}\rho_{\mathrm{RCP}}$, the H-GGA and DH-GGA functionals behaved similarly, giving a fairly constant value, around 0.008 a.u., although deviations for SOGGA11-X and especially for MN15 are visible. Quite the contrary, in the case of GGA, M-GGA, and especially HM-GGA functionals, the differences obtained are significant.

**Figure 7:**
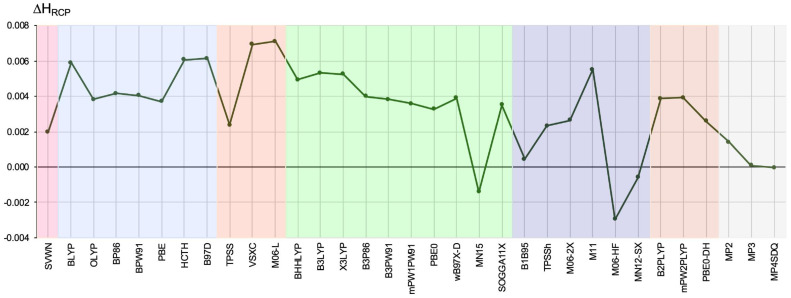
The difference (in relation to the CCSD method) in the value (in a.u.) of the total electronic energy density determined at the ring critical point of the 2,2-dichloro-1-(1H-pyrrol-2-yl)ethan-1-one molecule. The different types of methods are represented by a colored background: LDA—pink, GGA—blue, M-GGA—orangeish, H-GGA—green, HM-GGA—violet, DH-GGA—beige, perturbational methods—gray.

The results for $\Delta H_{\mathrm{RCP}}$ are shown in Figure 7.

As for $\Delta \rho_{\mathrm{RCP}}$ and $\Delta \nabla^{2}\rho_{\mathrm{RCP}}$, the scatter of the obtained $\Delta H_{\mathrm{RCP}}$ values is large. The largest errors in relation to CCSD were obtained by M06-L (0.0071 a.u.) and VSXC (0.0069 a.u.). Slightly smaller $\Delta H_{\mathrm{RCP}}$ errors (0.005–0.006 a.u.) were obtained for BLYP, HCTH, B97-D, B3LYP, X3LYP, and M11. On the contrary, the smallest deviations were obtained by functionals B1B95 (0.0004 a.u.) and MN12-SX (−0.0006 a.u.), which are the only ones that clearly outperformed the MP2 method (0.0014 a.u.). Thus, these two functionals should be considered worth using for QTAIM-based π-electron delocalization analysis [59]. It is worth noting that in the case of H-GGA functionals, as in the case of $\Delta \nabla^{2}\rho_{\mathrm{RCP}}$ (Figure 6), the MN15 functional clearly stands out, giving a negative, not a positive, deviation from the CCSD value. Moreover, even without this functional, the values of $\Delta H_{\mathrm{RCP}}$ are clearly more different than in the case of $\Delta \nabla^{2}\rho_{\mathrm{RCP}}$.

### 2.3. Testing of DFT Functionals for the Bond Delocalization Index

**Figure 8:**
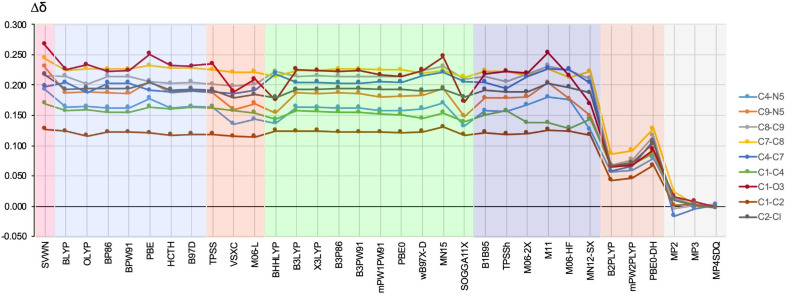
The difference (in relation to the CCSD method) in the value (in a.u.) of the delocalization index for the bonds shown on the right side of the figure. The different types of methods are represented by a colored background: LDA—pink, GGA—blue, M-GGA—orangeish, H-GGA—green, HM-GGA—violet, DH-GGA—beige, perturbational methods—gray.

Bond delocalization index (δ) is one of the most important QTAIM-based parameters because, as a quantity describing the number of electrons shared by two atomic basins, it is directly related to the bond order [60,61,62]. Moreover, it is strongly related to the exchange energy and therefore describes the covalent component of a bond [63,64]. The delocalization index differences determined for each method are presented in Figure 8.

Importantly, all the functionals gave positive Δδ values for all the bonds analyzed, thus indicating an overestimation of the obtained δ values relative to the reference values for CCSD. Thus, DFT gives too much covalent component of a bond. Nevertheless, in the group of the functionals tested, the double hybrids are by far the best. Namely, depending on the bond, the values of Δδ are in the range of 0.042–0.092 a.u. for B2-PLYP and mPW2-PLYP, and are somewhat greater for PBE0-DH (0.067–0.128 a.u.), while the Δδ values obtained by all the other functionals are much greater, from 0.116 a.u. to 0.267 a.u. As expected, the errors obtained by the MP2 method are even smaller (from −0.017 a.u. to 0.023 a.u.) than those for the DH-GGA functionals, while the values for MP3 and MP4SDQ are very close to zero.

As for the parameters $\Delta \rho_{\mathrm{BCP}}$, $\Delta \nabla^{2}\rho_{\mathrm{BCP}}$, and $\Delta H_{\mathrm{BCP}}$ previously discussed, the C=O bond is the most problematic (although not for all of the functionals tested). The greatest errors were obtained by SVWN (0.267 a.u.), M11 (0.253 a.u.), PBE (0.251 a.u.), and MN15 (0.247 a.u.), while the smallest were obtained by MN12-SX (0.168 a.u.), SOGGA11-X (0.172 a.u.), BHHLYP (0.175 a.u.), and VSXC (0.189 a.u.) (however, as mentioned, the DH-GGA functionals gave much smaller $\Delta \delta^{C=O}$ values). Equally problematic in describing a reliable δ value is the formally double C7–C8 bond of the imidazole ring (yellow line). This time, however, the errors obtained with all the functionals except the double hybrids are similar, roughly around 0.225 a.u.

The smallest errors Δδ with a fairly constant value of ca. 0.120 a.u., regardless of the functional used (but double hybrids, of course, with Δδ amounting to ca. 0.045 a.u. for B2-PLYP and mPW2-PLYP and 0.067 a.u. for PBE0-DH), were obtained for the single C1–C2 bond (brown line). This result shows that in terms of the reliability of the δ value obtained by DFT, a single nonpolar bond such as C–C is the least problematic.

From the results presented in Figure 8, it is clear that DH-GGA functionals significantly outperform the functionals from other rungs of the Jacob’s Ladder in terms of the reliability of the δ value. Of these, it is rather difficult to clearly find the best, although perhaps VSXC, BHHLYP, SOGGA11-X, and M06-2X can be recommended. Quite the opposite, in order to determine reliable δ values, the use of SVWN, PBE, MN15, and M11 can certainly be discouraged.

## 3. Crystal Structure and Methodology

### 3.1. Crystal Structure of 2,2-Dichloro-1-(1H-pyrrol-2-yl)ethan-1-one

The synthesis of 2,2-dichloro-1-(1H-pyrrol-2-yl)ethan-1-one [57] is detailed in Supplementary Materials (Figure S1), whereas the crystal data for 2,2-dichloro-1-(1H-pyrrol-2-yl)ethan-1-one (C${}_{6}$H${}_{5}$Cl${}_{2}$NO; *M* = 178.01 g/mol) shown in Figure 1 are as follows: triclinic, space group $P\bar{1}$ (no. 2), *a* = 4.5214(10) Å, *b* = 9.347(2) Å, *c* = 9.362(2) Å, α = 81.928(18)${}^{\circ }$, β = 78.065(18)${}^{\circ }$, γ = 77.212(17)${}^{\circ }$, *V* = 375.63(15) Å${}^{3}$, *Z* = 2, *T* = 123(1) K, μ(MoKα) = 0.788 mm${}^{-1}$, $D_{\mathrm{calc}}$ = 1.574 g/cm${}^{3}$, 5251 reflections measured (2.235${}^{\circ }\leq \Theta \leq$ 27.95${}^{\circ }$), 1612 unique ($R_{\mathrm{int}}$ = 0.0459), which were used in all calculations. The final *R*1 was 0.0304 [*I* > 2σ(*I*)] and wR2 was 0.0961 (all data). CCDC 2120678 contains the supplementary crystallographic data for this paper [65]. Detailed information on X-ray diffraction analysis can be found in Supplementary Materials (Tables S1 and S2 and Figure S2).

### 3.2. Methodology

The aim of this article is to test the exchange-correlation functionals of DFT in terms of their reliability of the description of the electron density distribution (EDD) in a real molecule. As already mentioned in the Introduction section, the reference structure is the high-quality X-ray crystal structure of 2,2-dichloro-1-(1H-pyrrol-2-yl)ethan-1-one (see Figure 1). This molecule has various types of bonds: nonpolar (e.g., C1–C2 and C1–C4), highly polarized (e.g., C1=O3 and C2–Cl14), single (e.g., C2–Cl14), double (C1=O3), and intermediate (e.g., N5–C4). This makes it possible to carry out the analysis also in terms of the type of bonding. Moreover, the presence of a ring makes it possible to study the influence of the functional selection on the EDD in it. Using this experimentally determined geometry, wave functions used to describe EDDs were determined utilizing the Gaussian 09 [66] and Gaussian 16 [67] programs.

The EDD of the 2,2-dichloro-1-(1H-pyrrol-2-yl)ethan-1-one molecule was probed by the most popular and useful QTAIM parameters characterizing bonds and rings, namely, the electron density (ρ), its Laplacian ($\nabla^{2}\rho$), and the total electronic energy density (*H*)—all computed at either the bond or ring critical point (marked as BCP or RCP, respectively) [54]. Importantly, as noted by Brémond et al. [50], the electron density at critical points well represents the quality of EDD of the whole molecular system. Additionally, the bond delocalization index (δ) was computed as well. All these parameters were obtained using the AIMAll program [68].

To benchmark exchange-correlation functionals, 30 of them were selected to represent all the rungs of Jacob’s Ladder [52,53]: SVWN [69,70], BLYP [71,72], OLYP [72,73], BP86 [71,74], BPW91 [75], PBE [76], HCTC [77], B97-D [78,79], TPSS [80], VSXC [81], M06-L [82], BHHLYP [83], B3LYP [71,72,84], X3LYP [85], B3P86 [74,84], B3PW91 [75], mPW1PW91 [86], PBE0 [87], ωB97X-D [88], MN15 [89], SOGGA11X [90], B1B95 [91], TPSSh [92], M06-2X [93], M11 [94], M06-HF [95], MN12-SX [96], B2-PLYP [97], mPW2-PLYP [98], and PBE0-DH [99]. The first of them, i.e., SVWN, represents the so-called local functionals (marked as LDA for local density approximation), which constitute the lowest rung of Jacob’s Ladder. Local functionals depend solely on the electron density. Then, BLYP, OLYP, BP86, BPW91, PBE, HCTH, and B97-D belong to the so-called gradient-corrected functionals, which depend not only on the electron density but also, additionally, on its gradient. They are usually abbreviated as GGA for generalized gradient approximation method. The next three functionals, i.e., TPSS, VSXC, and M06-L, belong to the group of meta-GGA (M-GGA) functionals, which also depend on higher derivatives of the electron density or, more commonly, on the kinetic energy density. Even higher, on the fourth rung of Jacob’s Ladder, there are very important hybrid (H-GGA) and hybrid-meta (HM-GGA) functionals, which are represented by the following functionals, respectively: {BHHLYP, B3LYP, X3LYP, B3P86, B3PW91, mPW1PW91, PBE0, ωB97X-D, MN15, SOGGA11-X} and {B1B95, TPSSh, M06-2X, M11, M06-HF, MN12-SX}. Hybrid functionals mix the exact exchange from the Hartree–Fock method [100] with the exchange and correlation from the GGA method. Finally, the highest rung of Jacob’s Ladder, i.e., the double hybrids (DH-GGA), which depend on virtual orbitals and therefore include nonlocal correlation, are represented by the B2-PLYP, mPW2-PLYP, and PBE0-DH functionals. A fairly large and reliable [4] aug-cc-pVQZ basis set [101,102,103,104,105] was used. It is of quadruple-ζ quality and contains diffuse functions to accurately describe lone electron pairs of chlorine atoms. The EDD obtained with each of these functionals was then compared with the EDD obtained at the CCSD/aug-cc-pVTZ level of theory, which was taken as a reference. The same method as a reference was used by Tognetti and Joubert [26] and most recently also by Brémond et al. [50]. This method belongs to the group of the so-called coupled cluster methods [106,107], and is only slightly inferior to the more computationally expensive CCSD(T) considered as “the gold standard” of computational methods [4]. In addition, computations were also made for the MP2 [108,109], MP3 [110,111], and MP4SDQ [112] perturbation theory methods. As for DFT, MP2 calculations were made with the aug-cc-pVQZ basis set, while, due to higher computational cost, aug-cc-pVTZ was used for MP3 and MP4SDQ. Reference values of the QTAIM-based parameters obtained at the CCSD/aug-cc-pVTZ level of theory are given in Table 1.

## 4. Conclusions

The problematic issue when performing computations using the DFT method is the selection of the appropriate exchange-correlation functional. Earlier theoretical studies show that there is no universal functional. Some functionals are better for some purposes and other functionals are better for others. To make matters worse, it follows that a given functional possibly produces acceptably good values for one parameter, while the values obtained for other parameters may have large errors. The worst, however, is when these parameters belong to the same group of physicochemical data; for example, they are the lengths of different bonds or the values of different angles in the same molecule.

The aim of this article was to investigate the reliability of various exchange-correlation functionals of the DFT method in terms of describing the electron density distribution. For this purpose, calculations of several fundamental parameters derived from QTAIM were performed and then compared to the reference values obtained by means of the CCSD method. These calculations were made using 2,2-dichloro-1-(1H-pyrrol-2-yl)ethan-1-one taken from the X-ray crystal structure determined for the purposes of this project.

The presented results show that the DFT method has a particular problem in reliably describing the electron density distribution in multiple and highly polarized bonds such as C=O, as well as conjugated bonds associated with large charge delocalization. In the case of 2,2-dichloro-1-(1H-pyrrol-2-yl)ethan-1-one, these are the bonds belonging to the imidazole ring and formally a single C–C bond connecting this ring to the –(CO)–CHCl${}_{2}$ group. On the contrary, description of the electron density distribution in single bonds proved to be much more reliable.

The obtained conclusions fit well with the current knowledge on the general behavior of exchange-correlation functionals. Namely, even for the considered QTAIM-based parameters, the functionals generally behave in an unsystematic way, and even if one gives good values for one parameter, it generally gives bad, or at least less reliable, values for another. This is quite a pessimistic side effect of using the DFT method. Moreover, this observation leads to the conclusion that nowadays it is much easier to discourage the use of certain functionals than to recommend some. The presented results show that the use of the M11 functional, in particular, should be discouraged for the purpose of a reliable description of the electron density distribution in molecules. In this respect, SVWN, BHHLYP, M06-HF, and, to a slightly lesser extent, BLYP, B3LYP, and X3LYP, also perform rather poorly. It should be emphasized here that in this group of nonrecommended exchange-correlation functionals there is B3LYP, which, however, is the most used. Therefore, its followers should seriously consider whether this functional really gives reliable results for the quantity they are concerned with, especially when these quantities are closely related to the electron density distribution in chemical bonds.

It is much more difficult to recommend a functional in terms of a reliable description of all the QTAIM-based parameters considered. The results show that none of the tested functionals stands out clearly in this respect. However, in the case of the electron density itself, being the most important QTAIM-based parameter, the VSXC, M06-L, SOGGA11-X, M06-2X, MN12-SX, and, to a slightly lesser extent, TPSS, TPSSh, and B1B95 can be recommended. It should be noted, however, that VSXC gave a very poor description of the electron density distribution in the ring critical point. On the other hand, the worst here was the M06-HF functional, which owes its worst position to the large error for the C=O bond. It was shown that functionals B1B95 and MN12-SX best describe the value of the total electronic energy density at the ring critical point and are therefore best suited for describing π-electron delocalization in an aromatic ring. Particularly noteworthy is the fact that double hybrid functionals clearly outperform other functionals in terms of a reliable value of the bond delocalization index, and, thus, the covalent component of a bond. It is noteworthy that all the functionals mentioned here turned out to be among the best in previous research [5,26,38,39,50].

It is also worth emphasizing that there is no clear overall improvement in the reliability of the description of the electron density distribution when climbing Jacob’s Ladder, i.e., from local functionals via gradient and meta-gradient to hybrid and double hybrid ones. The lowest-ranked local functionals in this hierarchy do, in many cases, yield unreliable results, but they may also produce fairly good or good results in other cases. On the contrary, theoretically, the best double hybrid functionals, although giving some of the most reliable results for many parameters (especially the bond delocalization index), fail in the case of others (e.g., the total electronic energy density at the bond critical point of the C=O bond). This result confirms the older conclusion of Medvedev et al. from 2017 [36] that the performance of the latest exchange-correlation functionals in terms of the proper description of the electron density distribution is still not satisfactory.

## Figures and Tables

**Table 1:** Reference values (in a.u.) of the QTAIM-based parameters obtained at the CCSD/aug-cc-pVTZ level of theory.

Property	Bond										RCP
	C4–N5	C9–N5	C8–C9	C7–C8	C4–C7	C1–C4	C1–O3	C1–C2	C2–Cl14	C2–Cl15	
ρ	0.2953	0.3172	0.3186	0.3059	0.3090	0.3021	0.3992	0.2453	0.1947	0.1909	0.0520
$\nabla^{2}\rho$	−0.7219	−0.6682	−1.0319	−0.9526	−0.9594	−0.9792	−0.3405	−0.6098	−0.2816	−0.2684	−0.0471
*H*	−0.4399	−0.4970	−0.3816	−0.3486	−0.3561	−0.3328	−0.7094	−0.2103	−0.1414	−0.1383	0.1767
δ	0.8968	0.9530	1.1614	1.1016	1.1222	0.9080	1.1287	0.7359	0.8636	0.8459	n/a

## Data Availability

Data available from the authors on reasonable request.

## Supplementary Materials

The following supporting information can be downloaded at: https://www.mdpi.com/article/10.3390/ijms232314719/s1. References [113,114,115,116,117] are cited in the supplementary materials.

## Abbreviations

The following abbreviations are used in this manuscript:

DFT	Density Functional Theory
EDD	electron density distribution
QTAIM	Quantum Theory of Atoms in Molecules
BCP	bond critical point
RCP	ring critical point
LDA	Local Density Approximation
GGA	Generalized Gradient Approximation method
M-GGA	meta-GGA functional
H-GGA	hybrid GGA functional
HM-GGA	hybrid M-GGA functional
DH-GGA	double-hybrid GGA functional
