# Supplementary material for: Testing of Exchange-Correlation Functionals of DFT for a Reliable Description of the Electron Density Distribution in Organic Molecules
Source: Int J Mol Sci. 2022 Nov 25;23(23):14719. doi: 10.3390/ijms232314719 (PMC9740346; doi:10.3390/ijms232314719)
Supplement: Supplementary file 1 [file ijms-23-14719-s001.zip › ijms-1973771-supplementary.pdf]

Testing of Exchange-Correlation Functionals of DFT for a Reliable Description of the Electron Density Distribution in Organic Molecules

SUPPORTING INFORMATION

Małgorzata Domagała [1], Mirosław Jabłoński [2*], Marcin Palusiak [1], Alina T. Dubis [3], Manfred Zabel [4], Arno Pfitzner [4]

[1] Faculty of Chemistry, University of Lodz, Pomorska 163/165, 90-236 Lodz, Poland
[2] Faculty of Chemistry, Nicolaus Copernicus University in Toruń, Gagarina 7, 87-100 Toruń, Poland
[3] Faculty of Chemistry, University of Białystok, Ciołkowskiego 1K, 15-245 Białystok, Poland
[4] Institute of Inorganic Chemistry, University of Regensburg, 93040 Regensburg, Germany
* Correspondence: teojab@chem.umk.pl (M.J.); Tel.: +48-056-611-4695

1.1. Synthesis of 2,2-dichloro-1-(1H-pyrrol-2-yl)ethan-1-one

The conventional Friedel-Crafts acylation method was applied for the preparation of aromatic ketone 2,2-dichloro-1-(1H-pyrrol-2-yl)ethan-1-one [1]. The title pyrroloketone was obtained using acid chlorides as acylating agents and stoichiometric amounts of $AlCl_3$ as a reaction promoter. Dichloroacetyl chloride (0.02 mol) was dissolved in 10 ml of anhydrous ethyl ether. The 0.02 mol of freshly distilled pyrrole was added dropwise and the reaction mixture was stirred for one hour at room temperature. After that time an aqueous solution of potassium carbonate (0.02 mol) was added. The resulting mixture was extracted twice with ether and the crude product was purified by crystallization from hexane.

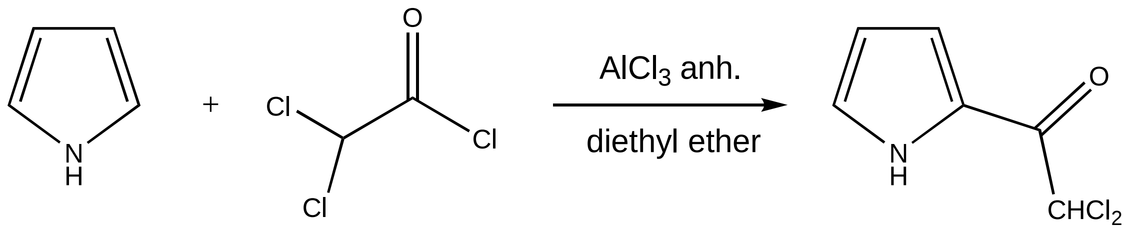

Figure S1. Reaction scheme.

1.2. X-ray diffraction analysis

The X-ray measurements of the 2,2-dichloro-1-(1*H*-pyrrol-2-yl)ethan-1-one were performed on a Stoe IPDS diffractometer with graphite monochromated MoKα radiation (λ=0.71073 Å). Data reductions were performed with Stoe IPDS [2] and the intensities were corrected for Lorentz and polarization effects. The

structure was solved by direct methods with SIR-97 [3] which revealed the positions of all non-H atoms. Non-H-atoms were refined anisotropically. All of the H-atoms were located in difference electron density map and their positions were allowed to refined together with individual isotropic temperature factors. Refinement of the structure was carried out on F^2 using full-matrix least squares procedures, which minimized the function $w(F^2_o-F^2_c)^2$ with SHELXL-2014/7 [4]. Geometry analysis and molecular plots were obtained using the PLATON program [5] and Mercury [6]. In Table S1 there are presented selected geometrical parameters between atoms in non-hydrogen skeleton of molecules.

Table S1. Selected geometrical parameters [Å, °] between atoms in studied structure.

N5-C9	1.357(2)	C4-N5-C9	109.1(2)
N5-C4	1.396(2)	N5-C4-C7	107.8(2)
C4-C7	1.411(2)	N5-C4-C1	120.8(2)
C7-C8	1.415(3)	C7-C4-C1	131.3(2)
C8-C9	1.397(3)	C8-C7-C4	106.5(2)
C4-C1	1.442(2)	C9-C8-C7	107.9(2)
C1-O3	1.233(2)	N5-C9-C8	108.8(2)
C1-C2	1.563(2)	O3-C1-C4	124.3(2)
C2-Cl4	1.773(2)	O3-C1-C2	120.4(2)
C2-Cl5	1.778(2)	C4-C1-C2	115.2(2)
		C1-C2-Cl14	111.3(1)
N5-H5	0.87(3)	C1-C2-Cl15	106.5(1)
C7-H7	0.94(2)	Cl14-C2-Cl15	110.6(1)
C8-H8	0.98(2)		
C9-H9	0.97(2)		
C2-H2	0.92(2)		

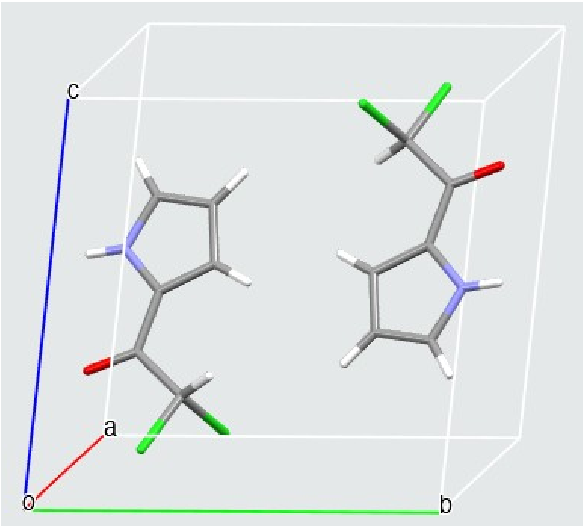

Figure S2. Unit cell packing of 2,2-dichloro-1-(1H-pyrrol-2-yl)ethan-1-one.

Table S2. Atomic coordinates of 2,2-dichloro-1-(1H-pyrrol-2-yl)ethan-1-one.

C	0.00675462	-0.00281860	-0.00322964
C	0.00675462	-0.00281860	1.55877036
O	1.07135960	-0.00281860	-0.62683102
C	-1.29569526	-0.03669697	-0.61882493
N	-1.43079866	-0.08894022	-2.00738999
H	-0.77314309	-0.09700230	-2.60402066
C	-2.59043433	-0.08499684	-0.05852820
C	-3.49432822	-0.17702025	-1.14290987
C	-2.74959993	-0.16886533	-2.32345552
H	-4.49002299	-0.15471130	-1.06744024
H	-3.00021123	-0.22517102	-3.27551479
H	-0.78733580	0.32854269	1.93845489
H	-2.80658169	-0.08381700	0.86830070
Cl	1.38127154	0.91352367	2.20317577
Cl	0.09869618	-1.70597545	2.06367917

[1] Nicolau, I.; Demopoulus, V.J. A Study of the Friedel-Crafts Acylation of 1-Benzenesulfonyl-1*H*-pyrrole in the Preparation of 3-Aroylpyrroles. *J. Heterocycl. Chem.* **1998**, *35*, 1345–1348.

[2] STOE IPDS-software, version 2.89, (1998). STOE & CIE GmbH, Darmstadt, Germany.

[3] Altomare, A.; Burla, M.C.; Camalli, M.; Cascarano, G.L.; Giacovazzo, C.; Guagliardi, A.; Moliterni, A.G.G.; Polidori, G.; Spagna, R. SIR97: A new tool for crystal structure determination and refinement. *J. Appl. Cryst.* **1999**, *32*, 115--119. DOI: 10.1107/S0021889898007717

[4] Sheldrick, G.M. Crystal structure refinement with SHELXL. *Acta Cryst.* **2015**, *C71*, 3--8. DOI: 10.1107/S2053229614024218

[5] Spek, A.L. Structure Validation in chemical crystallography. *Acta Cryst.* **2009**, *D65*, 148--155. DOI: 10.1107/S090744490804362X

[6] Macrae, C.F.; Sovago, I.; Cottrell, S.J.; Galek, P.T.A.; McCabe, P.; Pidcock, E.; Platings, M.; Shields, G.P.; Stevens, J.S.; Towler, M.; Wood, P.A. Mercury 4.0: from visualization to analysis, design and prediction. *J. Appl. Cryst.* **2020**, *53*, 226–235. DOI: 10.1107/S1600576719014092
